# Early-stage T1b adenocarcinoma arising in the remnant cystic duct after laparoscopic cholecystectomy: a case report and literature review

**DOI:** 10.1186/s12893-019-0647-9

**Published:** 2019-11-29

**Authors:** Pankaj Prasoon, Yuki Hirose, Jun Sakata, Kizuki Yuza, Kazuki Moro, Koji Toge, Kohei Miura, Masayuki Nagahashi, Takashi Kobayashi, Kouei Nihei, Atsuo Nakamura, Toshifumi Wakai

**Affiliations:** 1 Division of Digestive and General Surgery, Niigata University Graduate School of Medical and Dental Sciences, 1-757 Asahimachi-dori, Niigata City, 951-8510 Japan; 2 Department of Surgery, Tsubame Rosai Hospital, Tsubame, Japan; 3 Department of Gastrointestinal Medicine, Niigata Prefecture Yoshida Hospital, Tsubame, Japan

**Keywords:** Gallbladder neoplasms, Remnant cystic duct cancer, Adenocarcinoma, Early-stage, Haemobilia, Peroral cholangioscopy, Surgery

## Abstract

**Background:**

The cystic duct has been included in the staging classification scheme for gallbladder cancer since the 2010 publication of the AJCC Cancer Staging Manual (7th edition). To our knowledge, only seven other cases of adenocarcinoma arising in the remnant cystic duct following cholecystectomy have been reported in the English-language literature, and none has been reported as primary early-stage T1b remnant cystic duct cancer (CDC). We report, herein, a case of primary adenocarcinoma arising in the remnant cystic duct in a patient with history of laparoscopic cholecystectomy for gallstone disease.

**Case presentation:**

An 81-year-old female presented with abdominal pain. Her medical history included a laparoscopic cholecystectomy for cholecystolithiasis two years prior. Jaundice was observed; imaging studies suggested that this was caused by choledocholithiasis. Blood chemistry findings showed severe liver dysfunction. Endoscopic retrograde cholangiography revealed haemobilia from the common bile duct with no evidence of choledocholithiasis. A bile sample showed Papanicolaou class IV cytology. As the extent of tumour spread was undetermined by abdominal ultrasonography and endoscopic ultrasonography, peroral cholangioscopy (POCS) was performed, which revealed tiny papillary lesions within the confluence of cystic duct, and fine granular lesions in the centre of bile ducts, signifying early-stage remnant CDC. Extrahepatic bile duct resection with regional lymphadenectomy was done. Histopathological findings revealed a 42-mm tubular adenocarcinoma originating from the remnant cystic duct with the considerable shallow spread across the extrahepatic bile ducts. It invaded the fibromuscular layer, with no lymphovascular or perineural invasion, no lymph node metastasis (13 nodes examined), and uninvolved surgical resection margin (R0 resection), and was staged as pT1bN0M0, Stage I.

**Conclusions:**

Primary early-stage T1b remnant CDC is an uncommon condition for which early diagnosis is challenging; if intraoperatively recognized, it can complicate surgery. Our experience of this case and an overview of the English literature suggest that POCS is an efficient tool to diagnosis this tumour and assess its spread along the extrahepatic bile ducts.

## Background

The cystic duct has been included in the staging classification scheme for gallbladder cancer since the 2010 publication of the American Joint Committee on Cancer (AJCC) Cancer Staging Manual,7th edition [1]. Early diagnosis of cystic duct cancer (CDC) is difficult, and when discovered intraoperatively, this tumour can add to the surgeon’s challenge. The development of adenocarcinoma in the remnant cystic duct following cholecystectomy is uncommon, with only seven cases reported in the English-language literature (PubMed, National Library of Medicine, Bethesda, MD, USA), Google Scholar, Web of Science and Scopus up to March 2019 [2–8]. Here, we report an instance of primary adenocarcinoma developing within a remnant cystic duct and evaluate the eight documented cases (including the present case) to characterize the clinicopathological attributes of this rare tumour.

## Case presentation

**Fig. 1:**
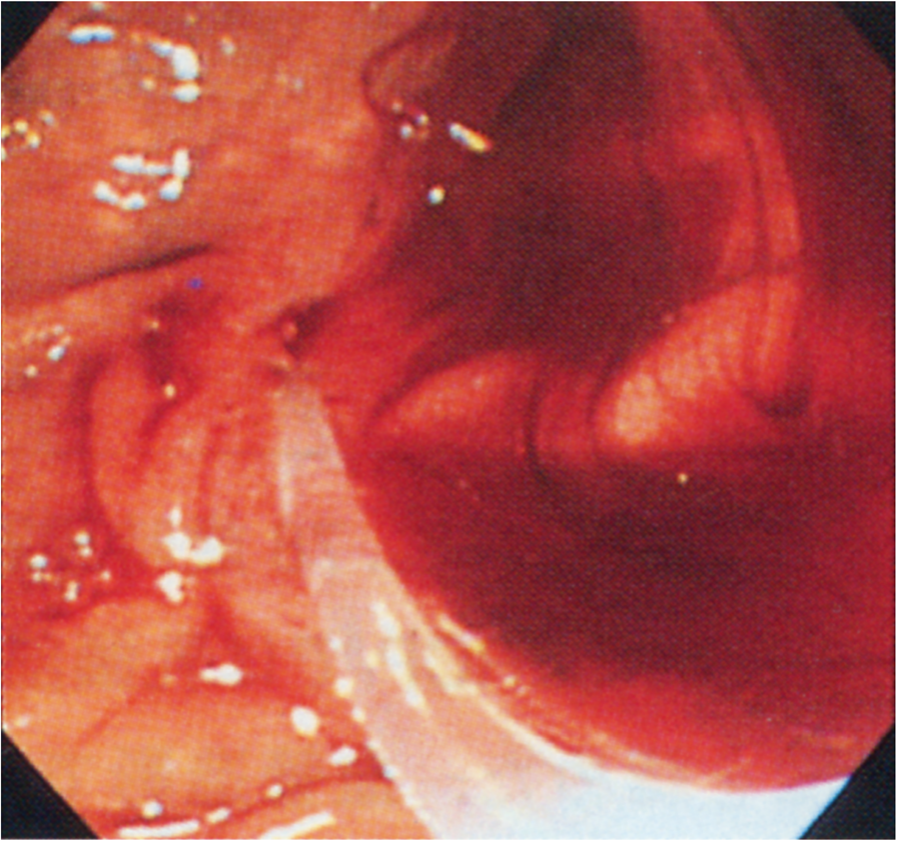
Endoscopic retrograde cholangiopancreatography shows haemobilia from the orifice of ampulla of Vater. (From Nakamura A, Yagi K, Sekine A, Nihei K, Oyamatsu M, Tamiya Y. An early cystic duct carcinoma with hemobilia: Report of a Case. Journal of Gastroenterological Imaging [in Japanese] 2002;4(5):593–7; with permission)

An 81-year-old female presented with acute abdominal pain. Her medical history included laparoscopic cholecystectomy for cholecystolithiasis two years prior. The histopathological report of the resected gallbladder specimen revealed chronic cholecystitis with no evidence of malignancy. The patient was jaundiced, and imaging studies suggested that it was caused by choledocholithiasis. Abdominal ultrasonography (US) revealed no dilatation of the intrahepatic bile ducts. Blood chemistry findings revealed significantly elevated transaminases and biliary enzymes, which indicated severe liver dysfunction (glutamic oxaloacetic transaminase: 2328 IU/L; glutamic pyruvic transaminase: 2454 IU/L; alkaline phosphatase: 475 IU/L; lactate dehydrogenase: 3348 IU/L; total bilirubin: 3.5 mg/dL). Serum tumour markers were all within normal limits (carcinoembryonic antigen: 1.7 ng/mL; carbohydrate antigen 19–9: 19 U/mL). Endoscopic retrograde cholangiopancreatography (ERCP) revealed haemobilia from the orifice of ampulla of Vater (Fig. 1) with no evidence of choledocholithiasis, suggesting that the jaundice was caused by haemobilia. She was discharged from hospital.

**Fig. 2:**
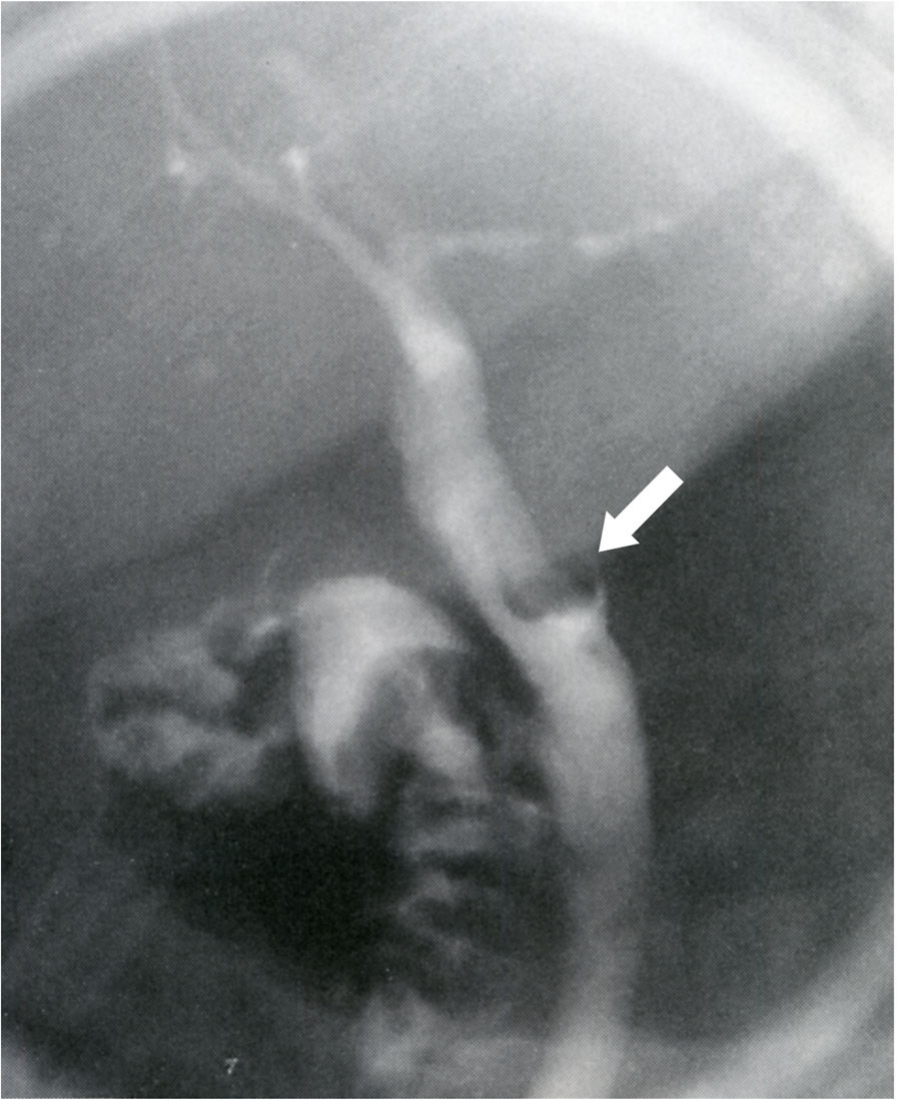
Endoscopic retrograde cholangiography depicts filling defect at the cystic duct. (From Nakamura A, Yagi K, Sekine A, Nihei K, Oyamatsu M, Tamiya Y. An early cystic duct carcinoma with hemobilia: Report of a Case. Journal of Gastroenterological Imaging [in Japanese] 2002;4(5):593–7; with permission)

**Fig. 3:**
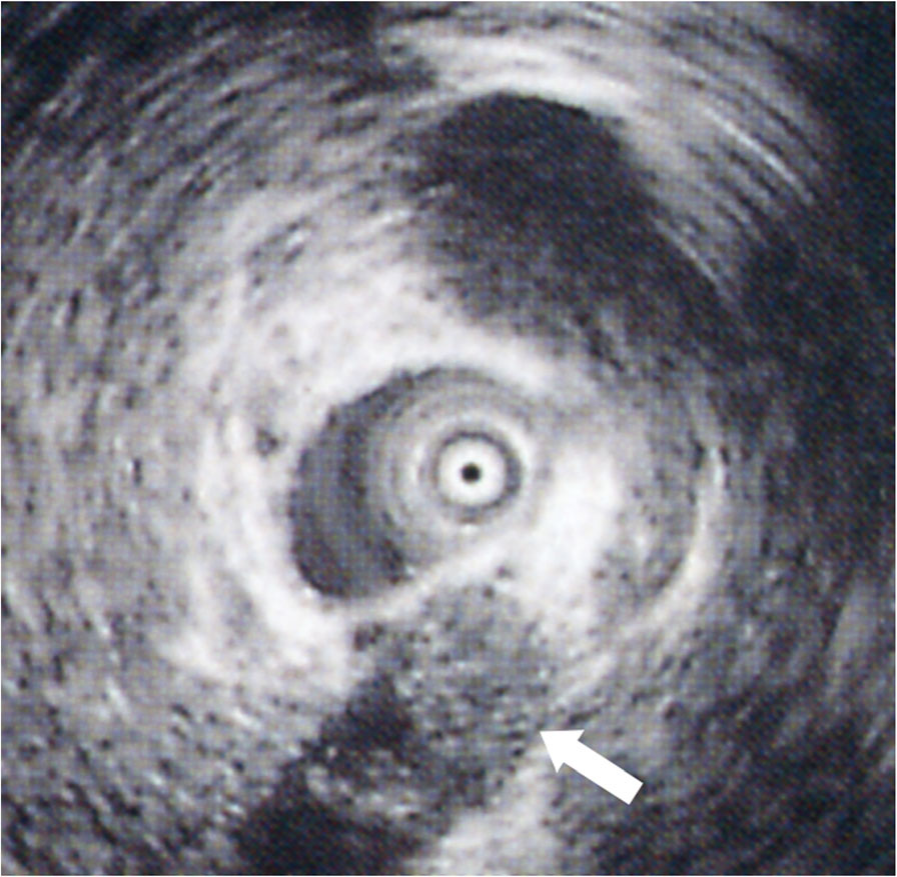
Endoscopic ultrasonography shows mass lesion in the remnant cystic duct. (From Nakamura A, Yagi K, Sekine A, Nihei K, Oyamatsu M, Tamiya Y. An early cystic duct carcinoma with hemobilia: Report of a Case. Journal of Gastroenterological Imaging [in Japanese] 2002;4(5):593–7; with permission)

**Fig. 4:**
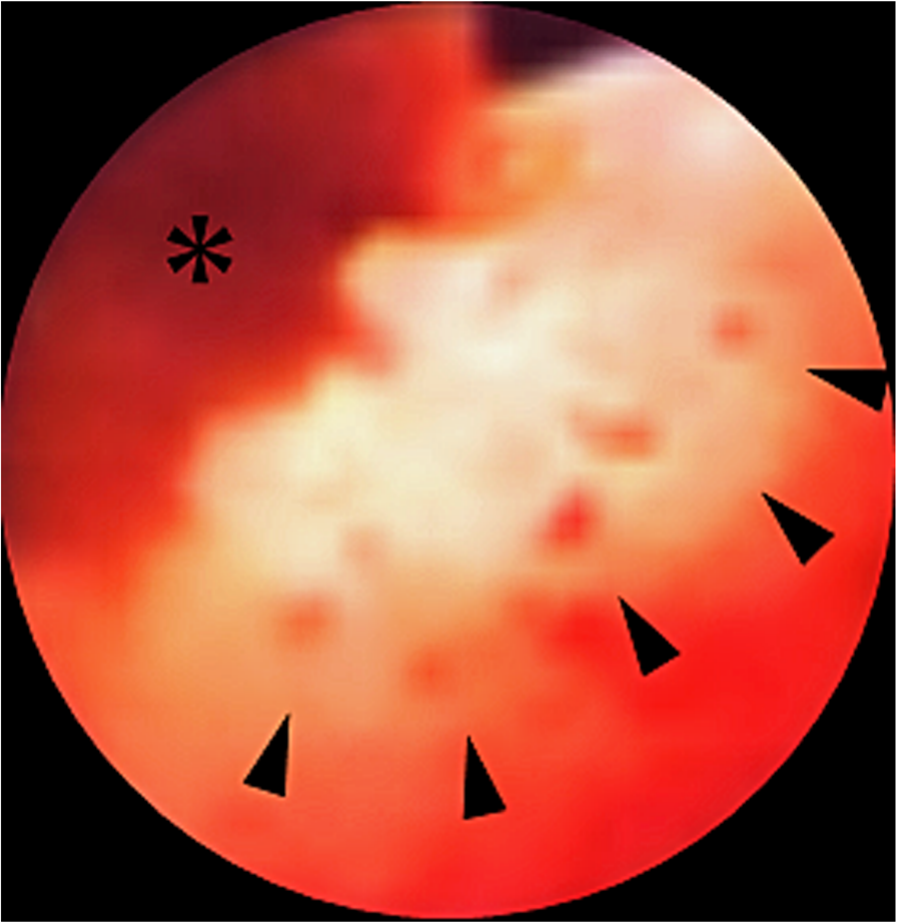
Peroral cholangioscopy depicts tiny papillary lesions (arrowheads) around the confluence of the cystic duct and bleeding point from tumour itself (*). (*From* Nakamura A, Yagi K, Sekine A, Nihei K, Oyamatsu M, Tamiya Y. An early cystic duct carcinoma with hemobilia: Report of a Case. Journal of Gastroenterological Imaging [in Japanese] 2002;4(5):593–7; with permission)

**Fig. 5:**
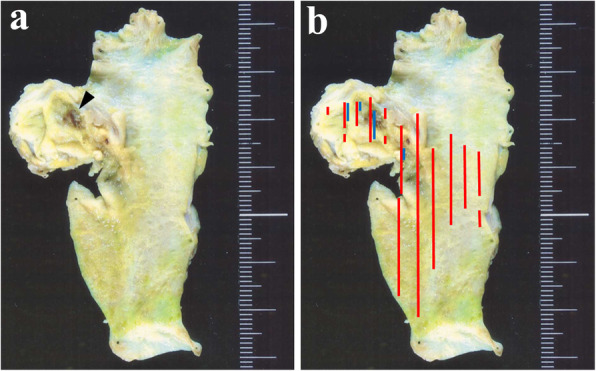
Resected specimen. **a** bleeding point (arrowhead) from the remnant cystic duct tumour. **b** red lines: area of T1 tumour; blue lines: area of tumour invasion of fibromuscular layer. (*From* Nakamura A, Yagi K, Sekine A, Nihei K, Oyamatsu M, Tamiya Y. An early cystic duct carcinoma with hemobilia: Report of a Case. *Journal of Gastroenterological Imaging* [in Japanese] 2002;4(5):593–7; with permission)

**Fig. 6:**
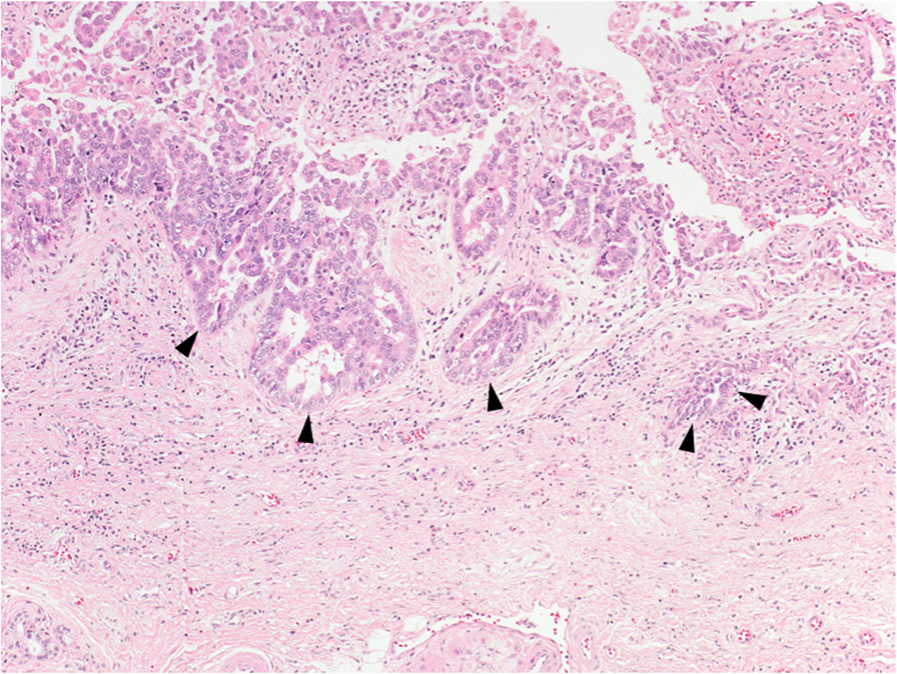
Adenocarcinoma invading fibromuscular layer (arrowheads)

At the outpatient department, 4 months after discharge, abdominal US found a 1-cm mass lesion at the union of cystic and common hepatic ducts. ERCP revealed a filling defect at the cystic duct (Fig. 2). Endoscopic US (EUS) discovered a lesion in the remnant cystic duct (Fig. 3). Bile samples taken during ERCP revealed Papanicolaou class IV cytology. The extent of tumour spread was undetermined by abdominal US and EUS, but peroral cholangioscopy (POCS) revealed tiny papillary lesions within the confluence of the cystic duct (Fig. 4) and fine granular lesions in the middle bile duct, which signified early-stage remnant CDC. She underwent an extrahepatic bile duct resection with regional lymphadenectomy; no residual tumour was found. A retrocolic Roux-en-Y loop of the jejunum was brought up and was anastomosed to the exposed right and left hepatic ducts separately (hepaticojejunostomy). Macroscopically, the cause of haemobilia was bleeding from the remnant cystic duct tumour (Fig. 5a). Histological inspection showed a well-differentiated tubular adenocarcinoma originating from the remnant cystic duct with the considerable shallow spread across the extrahepatic bile ducts (Fig. 5b), 42 × 40 mm in size, that had invaded the fibromuscular layer (Fig. 6), but showed no lymphovascular or perineural invasion, no lymph node metastasis (13 examined lymph nodes), and uninvolved surgical resection margin (R0 resection), and was classified as pT1bN0M0, Stage I, based on the AJCC Cancer Staging Manual, 8th edition [9]. The patient had an uneventful recovery and was discharged. She died of another cause (colon cancer) with no evidence of recurrent CDC, 4 years after the surgery for remnant CDC.

## Discussion and conclusions

Here, we present an uncommon entity, primary early-stage T1b adenocarcinoma arising in the remnant cystic duct following laparoscopic cholecystectomy. We reviewed the English-language literary works (PubMed, National Library of Medicine, Bethesda, MD, USA) published from January 1966 through March 2019, using the following Medical Subject Heading (MeSH) terms: “cystic duct” or [“surgery” and “gallbladder neoplasms”] in combination with “remnant cystic duct cancer”, “bile duct neoplasms”, “treatment outcome”, and/or “follow-up study.” References from the included articles were searched to identify additional cases and revealed that a total of seven patients who had undergone cholecystectomy suffered from subsequent remnant CDC following cholecystectomy [2–8]. To our knowledge, this is a first case in the English-language literature of primary early-stage T1b adenocarcinoma arising in the remnant cystic duct with a patient having history of laparoscopic cholecystectomy for gallstone disease.

In 1951, Farrar suggested diagnostic standards for CDC, which included (1) progression limited to within the cystic duct, (2) no neoplastic process in the gallbladder or hepatic or common bile duct, and (3) histological confirmation of malignant cells [10]; which the present case fulfils. In our case, tumour invasion was up to fibromuscular layer and limited within the remnant cystic duct (Fig. 5), the prior resected gallbladder specimen showed chronic cholecystitis with no evidence of malignancy, and histopathological findings provided a diagnosis of primary early-stage T1b adenocarcinoma arising in the remnant cystic duct.

Symptoms may appear more readily in early-stage remnant CDC than in gallbladder cancer. The cystic duct obstruction may cause symptoms like abdominal pain and jaundice as the gallbladder becomes distended, ultimately resulting in cholecystitis with fever, chills and inflammation [8]. Sakurai et al. [11] documented a case of early CDC, in which invasion was restricted to the fibromuscular layer; the papillary tumour projected into the common bile duct, remote from the junction of the cystic duct, to reach the common bile duct, causing obstructive jaundice. However, in the present case of a remnant CDC, the latter symptoms were jaundice and concomitant haemobilia, which was difficult to diagnose by conventional radiological modalities. In contrast, bleeding pseudoaneurysm of the cystic artery due to chronic cholecystitis treated by endovascular embolization and subsequent cholecystectomy have been well designated [12]. We noticed that the originating site of haemobilia was itself a tumour (Figs. 4 and 5 a). This is quite a rare phenomenon, with little published literature pertaining to this clinical feature.

Primary early-stage CDC is challenging to confirm, even in the twenty-first century. It may possibly characterize as a peril factor if it occurs as an intrusive neoplasm in the identical site. The cystic duct is pretty small and joins the gallbladder to the common bile duct. Its surfaces essentially incorporate four layers: mucosa (epithelium, lamina propria), muscle layer, perimuscular connective tissue, and serosa [1]. The perimuscular connective tissue has more substantial lymphatic vessels than do the shallower layers [13]. Due to their distinctive structure and site, carcinomas caused by the cystic duct are referred to as gallbladder carcinomas and therefore are thought to simply interfere with surrounding organs and lymph nodes [14]. Because of this, early-stage CDCs are leading-edge if they are identified. Early-stage CDC is very uncommon; documented cases account for only 0.03–0.05% of all autopsies [14]. We previously reported that POCS is an efficient diagnostic option for early detection of biliary tract cancer [15]. In the present case, POCS was useful in determining the superficial ductal spread of the cancerous area to achieve negative margins. We experienced that POCS is an efficient tool regarding diagnosis and determination of extent of tumour spread along the extrahepatic bile ducts.

Table 1 summarizes reported cases of remnant CDC. The extent of tumour spread included distant liver metastasis [3], tumours directly invading other adjacent organs, such as the pancreas [5], colon [6], and duodenum [7], and tumour invading to the main portal vein and hepatic artery [4], which indicates that detection of this tumour at later stages may lead to dismal outcomes. Do et al. [8] reported a case of adenocarcinoma, histopathologically diagnosed as pT2N0M0, that developed from the remnant cystic duct after cholecystectomy; its spread limited to the gallbladder viscus and the patient was alive 1 year after surgery with no recurrence.

**Table 1:** Summary of the reported cases of remnant cystic duct cancer [2–8]

Case no.	Author	Year	Cause of prior cholecystectomy	TI (yrs)	Surgical procedure	Histopathology and extent of tumor spread	Outcome (yrs)
1	Kuwayti et al. [2]	1957	Acute cholecystitis	3	Necropsy	ND	ND
2	Phillips et al. [3]	1969	Cholelithiasis	6	Amputation of tumor mass, T-tube drainage	Liver metastasis	ND
3	Dixon et al. [4]	1971	Chronic cholecystitis	5	Open biopsy, T-tube drainage	Involvement of the PV and RHA	ND
4	Gabata et al. [5]	2003	Cholecystolithiasis	0.5	PD	Involvement of the pancreatic duct	ND
5	Noji et al. [6]	2003	Cholelithiasis	15	ERH, BD, Co	pT3N2M0 Involvement of the transverse colon	0.5; NED
6	Eum et al. [7]	2008	Cholelithiasis	20	BD	Involvement of the duodenum, pN0	0.5; NED
7	Do et al. [8]	2014	Acute cholecystitis with gallstone	10	WR, BD after removal of the remnant cystic duct	pT2N0M0	1; NED
8	Present case	2019	Chronic cholecystitis with gallstone	2	BD	pT1bN0M0	4; DOO

*TI* time interval between prior cholecystectomy and surgery of remnant cystic duct cancer, *ND* not documented, *PD* pancreaticoduodenectomy, *ERH* extended right hepatectomy, *BD* resection of the extrahepatic bile duct, *Co* partial resection of the transverse colon, *WR* wedge resection of the gallbladder bed, *PV* portal vein, *RHA* right hepatic artery, *pTNM* pathological tumor-node-metastasis classification, *NED* (alive with) no evidence of disease, *DOO* died of other causes

Appropriate surgical treatment for remnant CDC is *en bloc* resection of the cystic duct, common bile duct, and regional lymphadenectomy, which could be the pillar of the treatment method. Among the eight reported cases (including the present case) in Table 1, early-stage (pT1bN0M0) remnant CDC was successfully recognized in our case, and the patient survived 4 years after its *en bloc* resection, with no evidence of recurrence. Thus, early detection and radical resection may improve outcomes in patients with remnant CDC. Primary early-stage T1b remnant CDC is an uncommon condition that cannot be easily diagnosed in its early stages. If serendipitously recognized during surgery, this tumour may greatly increase the difficulty of a procedure. Our understanding with this case and literature review indicates that POCS is an efficient tool for diagnosing this tumour and determining the extent its spread along the extrahepatic bile ducts. Wider study from multicentre institutes is needed to validate the long-term survival outcomes.

## Data Availability

The datasets utilized and/or reviewed throughout the present study can be obtained from the corresponding author on sensible request.

## Abbreviations

AJCC: American Joint Committee on Cancer
CDC: Cystic duct cancer
ERCP: Endoscopic retrograde cholangiopancreatography
EUS: Endoscopic ultrasonography
POCS: Peroral cholangioscopy
US: Ultrasonography
